# Human papillomavirus (HPV) and HPV vaccine awareness in Indigenous communities in Northwest Territories, Canada

**DOI:** 10.1371/journal.pone.0357586

**Published:** 2026-09-03

**Authors:** Afsaneh Omidimorad, Fariba Kolahdooz, Ghazal Kolahdooz, Adrian Wagg, Paul Veugelers, André Corriveau, Kami Kandola, Stephanie Irlbacher-Fox, Gina Ogilvie, Carolyn Gotay, Sangita Sharma

**Affiliations:** 1 Indigenous and Global Health Research Group, Department of Medicine, Faculty of Medicine & Dentistry, College of Health Sciences, University of Alberta, Edmonton, Alberta, Canada; 2 Division of Geriatric Medicine, Department of Medicine, Faculty of Medicine & Dentistry, College of Health Sciences, University of Alberta, Edmonton, Alberta, Canada; 3 Population Health Intervention Research Unit, School of Public Health, College of Health Sciences, University of Alberta, Edmonton, Alberta, Canada; 4 Independent Public Health Consultant, Yellowknife, Northwest Territories, Canada; 5 Department of Health and Social Services, Government of Northwest Territories, Yellowknife, Northwest Territories, Canada; 6 Hotıì ts’eeda Northwest Territories SPOR SUPPORT Unit, Yellowknife, Northwest Territories, Canada; 7 School of Population and Public Health, Faculty of Medicine, University of British Columbia, Vancouver, British Columbia, Canada; 8 Women’s Hospital and Health Centre, Vancouver, British Columbia, Canada; McGill University, CANADA

## Abstract

**Objectives:**

Human papillomavirus (HPV) is a common sexually transmitted infection linked to several cancers, including cervical cancer, which ranks fourth in cancer incidence and mortality among women globally. Despite its significance, awareness of HPV and the HPV vaccine remains limited across many populations. This project assessed HPV and HPV vaccine awareness among Indigenous adults in Northwest Territories communities in Canada and explored factors influencing HPV vaccine utilization; such data have never been available or explored.

**Methods:**

Using a community-based participatory research approach, quantitative and qualitative data were collected in 11 Indigenous communities in Northwest Territories. Indigenous adults aged 18 years and older were invited to complete a semi-structured questionnaire administered by trained local research assistants. Multiple logistic regression was used to examine potential associations among age, gender, education, and awareness of HPV and the HPV vaccine. A thematic analysis of qualitative data explored reasons for non-vaccination.

**Results:**

Among the 221 participants (66.5% women; mean age 43.6 years (±13.9)), approximately half had heard of HPV, and fewer than one-third had heard of the HPV vaccine. Education emerged as a key factor, with individuals with lower educational attainment being significantly less aware of HPV (p < .000). Age (p < .033), gender (p < .019), and education (p < .004) predicted vaccine awareness, with women, individuals with higher education, and younger adults being more likely to have heard about the vaccine. Only 26.3% of participants aged up to 26 years had received the HPV vaccine. Qualitative data revealed that limited awareness and insufficient information about HPV and the vaccine are the main barriers to vaccination. Participants recommended enhancing vaccine promotion by raising awareness about the connection between HPV and cancer, as well as increasing educational efforts in schools and communities.

**Conclusions:**

Limited awareness of the HPV vaccine may contribute to low vaccination rates. Tailored and targeted interventions are crucial to increasing HPV vaccine utilization in Indigenous communities in Northwest Territories.

## Introduction

Human papillomavirus (HPV) is a widespread sexually transmitted infection (STI) [1]. There are both low-risk variants of HPV, responsible for genital warts and non-malignant lesions, and high-risk variants, which are associated with cervical, anal, vulvar, vaginal, penile, and oropharyngeal malignancies. The incidence rates for HPV-related cancers can vary by gender and region [1]. Among men, oropharyngeal and anal cancers are the most common cancers related to HPV infection. Among women, cervical cancer is the most prevalent high-risk HPV-related cancer, accounting for 91.00% of such cases [2]. In Canada, between 1993 and 2018, 22,235 women were diagnosed with cervical cancer [3], and projections for 2024 estimated 1,600 new cases of cervical cancer, making it the fastest-increasing cancer among women in the country [4]. Overall, in Canada, the annual number of new cancer cases caused by HPV is expected to increase from 3,800–6,600 by 2042 [5]. Indigenous populations in Canada experience significantly higher rates of STIs, including HPV, which contributes to disproportionately high rates of cervical cancer [6], compared to non-Indigenous women. Indigenous women are up to 20 times more likely to develop cervical cancer and experience a fourfold higher mortality rate than non-Indigenous women [7].

In 2006, the World Health Organization (WHO) recommended HPV vaccination as the most effective strategy to reduce HPV-related disease incidence [8]. Several countries subsequently implemented HPV vaccination programs, primarily focusing on adolescent girls. In Canada, school-based vaccination programs for girls began in 2007–2008 [9]. Despite established evidence on the safety, quality, and efficacy of HPV vaccines [10], vaccination rates vary considerably within and between countries [11]. According to the 2021 Childhood National Immunization Coverage Survey, which used weighted data to estimate HPV vaccination coverage among Canadian children aged 14 years, 74.90% had received the HPV vaccine. Coverage was higher among girls (80.00%) than boys (69.90%) and was highest in Quebec (81.30%), followed by Ontario (74.00%) and other provinces/territories (72.10%). Urban residents (75.40%) had higher coverage than rural residents (72.10%). Notably, Indigenous children had significantly lower HPV vaccine coverage (55.80%) compared to non-Indigenous children (75.60%) [12].

In Northwest Territories (NWT), which accounts for nearly 14% of Canada’s landmass, 49.6% of the 41,070 residents are Indigenous [13]. Indigenous communities in Canada face barriers to accessing healthcare and health promotion interventions. These barriers include limited access to healthcare and health education systems, negative bias among healthcare professionals, and systemic racism and discrimination rooted in colonialism [14].

Studies indicate low levels of HPV awareness among Indigenous communities globally [15–17]. One of the first studies in Canada to examine HPV awareness among Indigenous communities was conducted in 2011 among Inuit women in Nunavik, Quebec and found that educational attainment was a key driver of HPV awareness in the population, though overall knowledge levels remained low [18]. Limited information about HPV and its vaccine, particularly its link to cervical and other HPV-related cancers, contributes to low vaccine utilization rates [17,19,20]. Socioeconomic status and ethnicity have been associated with lower HPV awareness [21–25], which may contribute to higher HPV infection and related disease rates among Indigenous communities [26].

To date, no project has assessed HPV or HPV vaccine awareness, or the factors influencing awareness and vaccine utilization, within Indigenous communities in NWT. In NWT, a publicly funded school-based HPV vaccination program was introduced in September 2009, initially for girls. The program has since expanded, and the nonavalent HPV vaccine (Gardasil®9) is now publicly funded for all eligible individuals aged 9–26 years, regardless of gender. [27,28]. Despite its availability, HPV vaccine utilization remains limited.

This project examined HPV and HPV vaccine awareness, utilization, and the factors influencing both within Indigenous communities in NWT. The findings from this project may help identify gaps and inform targeted interventions within Indigenous communities.

## Materials and methods

### Design and setting

This mixed-method project was implemented in 11 NWT communities varying in size, remoteness, and infrastructure between 2022 and 2024. Larger communities in the region (>1000 people) are connected by all-season roads and have hospitals and community health centers staffed by permanent nurses. Medium-sized communities (300–1000 people) also maintain year-round road access and have community health centers, though are without hospital facilities. Smaller communities (<300 people), in contrast, often lack all-season road connections, and only some are served by health centers. Regardless of size, all communities rely on the territorial hospital in Yellowknife for advanced care. The project employed a cross-sectional design and adhered to the community-based participatory research model, which emphasizes the active involvement of Indigenous communities as equal partners in all stages of the research process and fosters trusting relationships between the communities and researchers. This model also recognizes the importance of engaging community partners as experts regarding the historical, social, political, and cultural contexts that have a bearing on the research design and outputs, enhancing the potential for positive social change [29]. Accordingly, a Community Advisory Board (CAB), comprising Elders, local healthcare providers, youth, community representatives, government officials, and policymakers, was established to guide the project throughout its duration. The CAB met regularly with the research team through in-person meetings, including during visits to participating communities, as well as through ongoing email and telephone communication. The CAB contributed to the development and refinement of the project objectives and research questions, provided guidance on project design and implementation to ensure cultural relevance and alignment with community priorities, informed the interpretation of findings, and supported knowledge translation activities. Through an integrated knowledge translation approach, the CAB also helped facilitate the dissemination of project findings to participating communities and informed their application to policy and practice.

### Population and recruitment

A stratified, non-random sampling design was employed, with communities serving as the strata. Participants were self-identifying Indigenous adults (aged 18 years or older) who had resided in one of the communities for at least two months before data collection began. The required sample size was calculated for a single proportion at a 95% confidence level and 6% margin of error, assuming p = 0.5, yielding a target of 243 participants. This target was distributed proportionally across communities and further adjusted using finite population correction, and despite pandemic-related restrictions, approximately 91% of the expected participants were successfully recruited. With support from community partners, local research assistants (RAs) were hired and trained in each community to recruit and interview participants using convenience sampling methods. Recruitment methods were tailored to each community, including RAs contacting community members, social media posts, word of mouth, and passive advertisements in accessible community locations, such as local food stores and health centers.

### Data collection

Quantitative and qualitative data were collected through a semi-structured questionnaire administered by community RAs. The questionnaire underwent a rigorous development process and was reviewed by the CAB to ensure cultural sensitivity and clear, plain language. It was pilot tested to confirm that all questions were culturally relevant and easily understood by participants. The questionnaire was administered in English, with interpretation provided in the local language by local interpreters as needed.

The questionnaire included demographic questions on ethnicity, education level, and parental and employment status. Participants were asked to self-report both sex and gender. As responses to these two items were identical across participants, the use of either variable would not have altered subsequent analyses or findings. We elected to report gender throughout the manuscript in keeping with current HPV vaccination guidance and public health messaging, which have moved toward a sex- and gender-inclusive framing in recognition that HPV infection, prevention, and vaccination are relevant to individuals across the sex and gender spectrum and not limited to people with a cervix. HPV and HPV vaccine awareness were assessed by asking, “Have you ever heard of HPV?” and “Have you ever heard of the HPV vaccine?“ It also explored whether participants aged up to 26 years had received the HPV vaccine, as this age corresponds to the free vaccination eligibility in NWT [27]. Eligible participants who reported not receiving the vaccine were also asked open-ended questions about reasons for non-vaccination and suggestions to increase vaccine utilization.

Participants chose whether to complete the interview in person or by telephone. Each interview lasted approximately 15 minutes. Interviews were audio-recorded with participant permission. Responses to open-ended questions were transcribed verbatim, and transcriptions were cross-checked with audio recordings for accuracy. All participant responses were entered into online forms developed using REDCap (version 8.1.1). Each participant received a $75.00 gift card redeemable at local grocery stores as an honorarium.

### Compliance with ethical standards

Research with Northern Indigenous communities requires extensive preparation to facilitate respectful and collaborative engagement. Before initiating a project, investigators conduct in-person consultations with each community, often before submitting funding applications. This preparatory stage is resource-intensive and logistically challenging, due to the remoteness of many communities, unpredictable weather, and the high costs of travel and accommodation. In addition to institutional ethics approval, researchers must obtain a research licence, contingent upon negotiated agreements on data stewardship, memoranda of understanding, and financial commitments with communities and partner organizations. Licence approval typically requires formal endorsement from multiple Indigenous communities and organizations, thereby ensuring that proposed research activities are reviewed and authorized across several levels of governance. Ethical approval for this project was granted by the University of Alberta's Research Ethics Board (Pro00102250), and a research licence was issued by the Department of Education, Culture, and Employment of the Government of the Northwest Territories (GNWT). To support collaboration, research agreements and memoranda of understanding were signed with the participating communities, the GNWT Department of Health and Social Services, and Northwest Territories Health and Social Services Authority. Participants received the informed consent form before the interview. Local research assistants were available to ensure participants understood the project aims and the informed consent process. Written informed consent was obtained before in-person interviews. For telephone interviews, the consent form was read aloud, verbal informed consent was obtained before the interview began, and the consent type was documented in REDCap by the interviewer. Project findings were shared with participating communities through community presentations.

### Data analysis

Quantitative data analyses were performed utilizing SAS statistical software (SAS Version 9.4, SAS Institute Inc., Cary, NC). The proportions of participants who were aware of HPV and the HPV vaccine were calculated. Prior to multivariable logistic regression to examine potential associations among age, gender, education, parental status, work status, and awareness of HPV and the HPV vaccine, collinearity among candidate predictors was assessed, examining variance inflation factors (VIFs), tolerance values, and condition indices. Thresholds of VIF > 5.0, tolerance < 0.10, and condition index > 30 were applied to identify problematic collinearity. Confounding was evaluated using the change-in-estimate method: crude binary logistic regression models were fitted separately for each predictor, followed by a fully adjusted model including all predictors simultaneously. A change of ≥10% in the odds ratio between crude and adjusted models was used to identify confounding. Variables identified as confounders were retained in the final model. Results are presented as odds ratios (ORs) with corresponding 95% confidence intervals (CIs). All tests were two-sided, and p-values of 0.05 were considered statistically significant.

For qualitative data, a thematic analysis approach was utilized. Two researchers independently reviewed the data multiple times, systematically coded them, and organized the codes into themes through an inductive analytical process.

## Results

A total of 221 Indigenous community members participated in this project, with 120 participating in person and 101 by phone. The main indigenous identity (78.44%) was “First Nations”. Most participants (66.21%) were younger than 50 years old (n = 145), with a mean age of 43.60 years (±13.90), and 66.51% were women (n = 145). Regarding education, 57.14% of participants had completed some high school education (n = 124), while 24.88% had completed post-secondary education (n = 54). In terms of employment, 55.25% of participants reported being employed for pay (n = 121) (Table 1).

**Table 1:** Demographic characteristics of participants in NWT (n = 221).

Variables^a^	Frequency n (%)
**Indigenous identity**	
First Nations	171 (78.44)
Métis	15 (6.88)
Inuit	32 (14.68)
**Age (years)**	
19-29	36 (16.44)
30-49	109 (49.77)
50-65	60 (27.40)
>65	14 (6.39)
**Gender**	
Woman	145 (66.51)
Man	73 (33.49)
**Education level**	
Elementary school	10 (4.61)
Middle school	29 (13.36)
High school	124 (57.14)
Post-secondary	54 (24.88)
**Work for pay**	
Yes	121 (55.25)
No	98 (44.75)
**Parent of child 9–18 years**	
Yes	102 (49.27)
No	105 (50.73)

^a^Missing data were not included in the calculation of the proportions. Missing data for all variables were less than 5 except parents (missing = 14).

Regarding awareness of HPV and the HPV vaccine, 49.52% (n = 103) of participants reported having heard of HPV, and 29.95% (n = 62) reported awareness of the HPV vaccine (Table 2).

**Table 2:** Awareness of HPV and the HPV among adults in NWT (n = 221)^a^.

	Heard about HPV n (%)	Heard about the HPV vaccine n (%)
**Yes**	103 (49.52)	62 (29.95)
**No**	100 (48.08)	141 (68.12)
**Prefer not to answer**	5 (2.40)	4 (1.93)

^a^Missing data were excluded from the analysis. Missing data for Heard of HPV = 13, Heard of HPV vaccine = 14.

Before conducting the multiple logistic regression analysis, multicollinearity among the predictor variables (education level, age group, gender, parental status, and work status) was assessed. Although some pairwise associations between predictors were statistically significant in chi-square analyses, multicollinearity was not considered problematic. Variance inflation factors (VIFs) ranged from 1.13 to 1.75, tolerance values were all greater than 0.88, and the maximum condition index was 6.97, below the commonly accepted threshold of 30. These findings indicated that the predictor variables could be included simultaneously in the logistic regression model. Crude logistic regression models were fitted separately for each predictor and compared to the fully adjusted model. All four variables showed OR changes exceeding the 10% change threshold, indicating that each variable confounded the others. All four predictors were therefore retained in the final multivariable model. In the multiple logistic regression, education level emerged as a significant predictor of having heard of HPV. Individuals with elementary/middle school education (OR=0.09; 95% CI: 0.02–0.32; p < .000), and individuals with high school education (OR=0.36; 95% CI: 0.16–0.80; p < .005), were significantly less likely to be aware of HPV compared to participants with post-secondary education. Also, age, gender, and parental and employment status were not predictors of HPV awareness after adjustments (Table 3).

**Table 3:** Proportion of participants’ awareness of HPV/HPV vaccine and association between general characteristics among adults in NWT (n = 203)^a^.

	Heard of HPV^a^			Heard of the HPV vaccine^a^		
Variables	Yes n (%)	No n (%)	OR^b^ (95% CI)	Yes n (%)	No n (%)	OR^b^ (95% CI)
**Age groups^a^**						
>50	26 (37.68)	43 (62.32)	1.0	9 (13.43)	58 (86.57)	1.0
30-49	54 (54.55)	45 (45.45)	0.94 (0.41-2.166)	41 (40.59)	60 (59.41)	3.23 (1.24-8.43)
19-29	23 (65.71)	12 (34.29)	2.26 (0.84-6.10)	12 (34.29)	23 (65.71)	3.77 (1.14-12.41)
p-value			**<**0.172			**<0.033**
**Gender^a^**						
Woman	74 (54.81)	61 (45.19)	1.0	52 (38.81)	82 (61.19)	1.0
Man	28 (43.08)	37 (56.92)	0.92 (0.45-1.87)	9 (13.64)	57 (86.36)	0.34 (0.14-0.84)
p-value			**<**0.831			**<0.019**
**Education^a^**						
Post-secondary	39 (76.47)	12 (23.53)	1.0	31 (59.62)	21 (40.38)	1.0
High school	57 (50.44)	56 (49.56)	0.36 (0.16-0.80)	25 (21.93)	89 (78.07)	0.26 (0.11-0.58)
Elementary/Middle school	5 (14.71)	29 (85.29)	0.09 (0.02-0.32)	5 (15.15)	28 (84.85)	0.40 (0.08-0.91)
p-value			**<0.000**			**<0.004**
**Parent of a child 9-18 years**						
Yes	62 (61.39)	39 (38.61)	1.0	43 (42.57)	59 (57.43)	1.0
No	40 (39.60)	61 (60.40)	0.54 (0.23-1.08)	18 (30.20)	83 (69.80)	0.51 (0.21-1.19)
p-value			<0.079			<0.121
**Employed**						
Yes	69 (67.65)	33 (32.35)	1.0	44 (39.29)	68 (60.71)	
No	45 (45.00)	55 (55.00)	0.68 (0.34-1.34)	17 (18.89)	73 (81.11)	0.54 (0.26-1.29)
p-value			**<**0.269			**<**0.185

^a^Participants who preferred not to answer and missing data are excluded from the analysis. Missing data for age = 2, gender = 3, education = 4, Heard of HPV = 13, Heard of HPV vaccine = 14. Prefer not to answer for Heard of HPV = 5, Heard of HPV vaccine = 4.

^b^Adjusted odds ratio for other variables.

For HPV vaccine awareness, age, gender, and education level were significant predictors. Individuals aged 30–49 years (OR=3.23; 95% CI: 1.24–8.43; p < .028) and 19–29 years

(OR=3.77; 95% CI: 1.14–12.41; p = .016) were more likely to be aware of the HPV vaccine than

participants over 50 years. Men were significantly less likely to be aware of the HPV vaccine than women (OR=0.34; 95% CI: 0.14–0.84; p < .019). Participants with elementary/middle school education (OR=0.40; 95% CI: 0.08–0.91; p = .042) and participants with high school education (OR=0.26; 95% CI: 0.11–0.58; p < .001) were less likely to be aware of the HPV vaccine compared to community members with post-secondary education (Table 3). Employment and parental status were not associated with HPV vaccine awareness after adjustments.

Among people aged up to 26 years (n = 19), 26.32% reported receiving the HPV vaccine (n = 5), 57.89% reported not receiving it (n = 11), and 15.79% were unsure of vaccination status and preferred not to answer (n = 3).

### Qualitative results

Among participants aged 19–26 years who were asked about HPV vaccination status, 11 individuals reported not having received the HPV vaccine. The participants provided responses to an open-ended question about reasons for non-vaccination. Two main themes emerged from the reasons participants shared: awareness of and access to information about the HPV vaccine, and vaccine confidence. Participants also offered suggestions to improve HPV vaccine utilization.

#### Theme 1: Awareness and availability of information on the HPV vaccine.

Some participants expressed unfamiliarity with HPV and the vaccine and explained that inadequate dissemination of information about the HPV vaccine’s availability influenced the decision not to receive it.

“*I don't even know what it is, to be honest; this is the first time I have even heard of it.”*

*“Nothing stopped me. I just didn’t know it is available here”.*

Another participant noted the need for more information to feel confident in making the decision, stating, “*I don't think I researched it enough to feel comfortable with getting it at the time when I was offered it, so I didn't feel comfortable taking it without doing specific research for it.”*

#### Theme 2: Vaccine confidence.

One participant discussed vaccine hesitancy shaped by familial attitudes and personal reluctance, sharing, *“Honestly, I feel like when it was offered to me, I was still living with my family, and my mom did not really want me to get it. So, I have, like, that has a big reason why I didn't get it, and then after that, I just wasn't really interested in researching it.”*

#### Suggestions regarding how to improve HPV vaccine utilization.

Participants provided several recommendations to improve HPV vaccine utilization, emphasizing the importance of information dissemination and awareness-raising efforts. Suggestions included highlighting the link between HPV and cancer, promoting vaccine awareness in schools, and increasing the availability of informational materials in communities.

One participant recommended, *“Let them know that it can turn into cancer down the road.”* Another participant suggested school-based promotion, recalling, *“Getting it promoted. I have never seen it promoted at all, and I think they did a good job when I was in school to get the parents to sign the form, and then the whole class goes.”*

## Discussion

The goal of this project was to assess awareness of HPV and the HPV vaccine within Indigenous communities in NWT and to identify factors that may influence HPV vaccine utilization. Half of the participants in this project were aware of HPV, whereas less than one-third were aware of the HPV vaccine. While there is significant variation in awareness regarding HPV and the HPV vaccine globally [22,30–32], and across adults in Canada [33], several studies have found that awareness of HPV is similarly low among Indigenous communities around the world [15,16,18]. However, there are few studies on Indigenous communities' awareness about HPV in high-income countries. In Canada, such low levels of awareness regarding HPV may contribute to the higher levels of HPV infection observed among Indigenous communities [7]. This is further compounded by lower rates of cervical cancer screening, which contribute to higher rates of cervical cancer [5,34]. Factors that may contribute to these limited levels of awareness, including socioeconomic status [21], limited access to healthcare as a primary source of health information [6,22], and sensitivities surrounding sexual health promotion [15], warrant further investigation.

In this project, gender was found to be a factor in HPV vaccine awareness, with women demonstrating higher levels of awareness than men. Previous research on gender and awareness of the HPV vaccine has similarly found that women demonstrate higher levels of awareness and knowledge compared to men [35,36]. Exposure to reproductive health services (e.g., menstrual health and contraceptive care), female-focused informational campaigns, and vaccination programs primarily directed at women may explain higher HPV and HPV vaccine awareness among women.

The results showed that people aged under 50 were more likely to be aware of the HPV vaccine than other age groups, which aligns with other studies which have reported higher HPV awareness among younger women [30,33] Another study conducted in the United States found that parents with a child approaching or within the recommended HPV vaccination age were more likely to have heard of HPV vaccines [37]. In our project, parental status was associated with HPV vaccine awareness in the unadjusted analysis; however, after adjusting for other covariates in the multivariable model, this association was no longer statistically significant.

In this project, post-secondary education was associated with greater awareness of HPV and the HPV vaccine. Previous studies have reported variation in HPV awareness by education level. A study among Indigenous women in Nunavik, Canada, showed HPV awareness was significantly associated with having 13 or more years of education [18]. A United States study found that HPV awareness varied from 34.7% among adults with less than a high school education to 74.7% among people with a college degree or higher [22]. A study in Canada similarly found that individuals with a university or college education exhibited nearly twice the level of knowledge regarding HPV compared to individuals without a college education [38]. Post-secondary education may offer greater opportunities to learn about health-related topics, increasing awareness of health issues and preventative care measures [39], including HPV and the HPV vaccine.

Although previous studies suggest that vaccine hesitancy is a major barrier to receiving the HPV vaccine [40–42], qualitative findings from this project indicate that limited awareness of HPV and restricted access to vaccine information are primary determinants of low HPV vaccine utilization.

Participants’ suggestions for improving HPV vaccine utilization included increasing awareness of and disseminating information about the HPV vaccine. Consistent with prior research, these findings emphasize the importance of improving HPV knowledge and awareness to promote HPV vaccine utilization [33,43,44]. Collaborative efforts with Indigenous communities are essential to develop culturally relevant health information initiatives that engage Elders, parents, and youth and to increase HPV vaccine acceptance in Indigenous communities in Canada and beyond. By fostering intergenerational dialogue, engaging trusted community leaders, and addressing cultural and gender-specific concerns, these initiatives may help normalize vaccination, support informed consent, and empower families and individuals to take ownership of health decisions [42,45]. Awareness initiatives targeting men and individuals with lower levels of education are also warranted.

This project adds to the limited evidence on HPV and HPV vaccine awareness among Indigenous communities in NWT. Notably, the research process involved Indigenous communities throughout. Participants were primarily women, educated, and may not be representative of the general population. Given that the HPV vaccine in Canada is primarily delivered through school-based programs targeting adolescents and teenagers, lower levels of awareness among participants aged 19 years and older may be expected. However, adults in this age group often play an important role in health decision-making for children and adolescents, including vaccination decisions. Therefore, there is an expectation that adults over 19 years may have some awareness of the vaccine through exposure to school-based immunization programs for children. Future research should include a more diverse range of participants to better understand the factors influencing HPV vaccine awareness and utilization within Indigenous communities.

The results of this project indicate that limited awareness of HPV and the HPV vaccine, particularly among men and individuals with lower levels of education, is a key factor contributing to low HPV vaccine utilization within Indigenous communities in NWT. To improve HPV vaccination, it is crucial to prioritize the dissemination of tailored information and the implementation of community-based awareness initiatives.
